# Primary closure combined with C-tube drainage through cystic duct after laparoscopic common bile duct exploration is safe and feasible for patients

**DOI:** 10.3389/fsurg.2022.972490

**Published:** 2022-10-25

**Authors:** Xin Sui, Zhenghui Sui, Xingwei Gu, Mingjin Ding, Ganggang Miao

**Affiliations:** ^1^ Department of General Surgery, The People's Hospital of Danyang, Affiliated Danyang Hospital of Nantong University, Danyang, China; ^2^ Department of General Surgery, Affiliated Nanjing Hospital of Nanjing Medical University, Nanjing, China

**Keywords:** c-tube drainage through cystic duct, t-tube drainage, primary closure, laparoscopic common bile duct exploration, choledocholithiasis

## Abstract

**Objective:**

Biliary duct management is of great significance after laparoscopic cholecystectomy (LC) combined with laparoscopic common bile duct exploration (LCBDE) in the treatment of cholecystolithiasis accompanied with common bile duct (CBD) stones. This study is to evaluate the safety and effectiveness of primary closure with C-tube drainage through cystic duct after LC + LCBDE.

**Methods:**

Through a retrospective study, 290 patients who underwent LC + LCBDE in our hospital from January 2019 to April 2022 were enrolled and divided into 2 groups. 143 patients underwent primary closure with C-tube drainage through cystic duct (C-tube group) and the other 147 patients underwent traditional T-tube drainage (T-tube group). Personal information, perioperative examinations, surgical results, and follow-up results were collected and analyzed.

**Results:**

There were no significant differences in the average age, gender, the mean of CBD diameters and the rate of comorbidities (acute cholecystitis, obstructive jaundice, acute pancreatitis and acute cholangitis) between the two groups (*P* > 0.05). Hospital stay, postoperative hospital stay were significantly shorter in the C-tube group than T-tube group (*P* < 0.05). In addition, the average time of placing and removal the drainage tubes was significantly less than those of the T-tube group (*P* < 0.05). This study also showed significant differences in the incidence of postoperative abdominal infection and soft tissue infection in the two groups (*P* < 0.05). There were no significant differences in the incidence of postoperative complications including cholangitis, bile duct stenosis, mortality in two groups. There were also no significant differences between the two groups of the recurrence of CBD stones, reoperation and readmition in 30 days during the median follow-up of 6 months.

**Conclusions:**

Compared with T tube drainage, patients with C-tube drainage after LC + LCBDE with primary closure of cystic duct recovered faster and had fewer complications. C-tube drainage is a safe and feasible treatment option for patients with cholecystolithiasis and choledocholithiasis.

## Introduction

Cholecystolithiasis is one of the most common clinical diseases, and 10%–15% of the patients with cholecystolithiasis have common bile duct (CBD) stones concurrently (1). Timely removal of common bile duct stones is very important to avoid a series of complications such as acute cholangitis, acute pancreatitis and secondary liver injury. Laparoscopic cholecystectomy (LC) is a consensus of treating cholelithiasis nowdays, what we call the “gold standard" (2). Currently, there are controversial views about the laparoscopic treatment of choledocholithiasis. Treatments of choledocholithiasis have undergone different stages of development and improvements, the universal therapies include laparoscopic common bile duct exploration (LCBDE), endoscopic retrograde cholangiopancreatography (ERCP), endoscopic sphincterotomy(EST), etc (3). Williams E, et al. (4) had proposed in the guidelines for the management of CBDS that LCBDE and ERCP were both very successful in removing CBDS. We carried out a study on LC + LCBDE by considering that LC + LCBDE can avoid the sequelae of endoscopic sphincterotomy, such as duodenal papilledema, stenosis, perforation, bleeding (5, 6).

Traditionally, T-tube drainage following LCBDE has been the standard treatment of choledocholithiasis (7). With the development of laparoscopic technique, surgeons are more inclined to place T-tube drainage during LCBDE in recent years. Nevertheless, some postoperative complications of T-tube drainage can not be avoided. The most common complications include bile leakage, hemorrhage, and bile duct stenosis. It is worth noting that bile leakage can cause inflammation of the bile duct and surrounding tissues, and can cause biliary peritonitis (8). Many medical centers are exploring more minimally invasive treatment methods to treat choledocholithiasis, they especially focus on placing tubes for bile duct drainage. Recent researches show primary closure with knotless barbed sutures or with D-J tube drainage are available (9, 10).

In this retrospective cohort study, we analyzed the patients' basic information, perioperative examinations, surgical outcomes and follow-up results in order to assess the effectiveness and feasibility of C-tube drainage through cystic duct following LC + LCBDE.

## Methods

### Patient selection

From January 2019 to April 2022, totally 290 patients underwent LC + LCBDE were enrolled in this study and divided into 2 groups. 143 patients in C-tube group underwent primary closure with C-tube drainage, the other 147 patients in T-tube group underwent T-tube drainage. Cholecystolithiasis accompanied with Choledocholithiasis was diagnosed and confirmed by preoperatively B-type ultrasonography (BUS), computed tomography (CT), magnetic resonance cholangiopancreatography (MRCP) or intraoperative Cholangiogram (IOC) (9). Relative personal and hosptalized information, surgical outcomes, postoperative complications and follow-up results of the different groups were compared. This study was approved by the ethics committee of affiliated Danyang Hospital of Nantong University. Each patient was informed of the condition and signed the informed consent for surgery.

The inclusion criteria of this study: (1) CBD diameter ≥8 mm as shown by preoperative BUS, CT, MRCP or IOC; (2) preoperative confirmation of CBD stones using BUS, CT,MRCP or IOC; (3) sphincter of Oddi in good condition. The exclusion criteria: intrahepatic multiple stones, hepatolithiasis, Mirizzi syndrome, gallbladder carcinoma or bile duct carcinoma, a history of upper abdominal surgery.

### Operative techniques

All operations are performed by the attending surgeons in the same department in accordance with standard principles. The patient was placed in a supine position and underwent transtracheal general anesthesia. An inflatable puncture needle was inserted through the umbilical incision to set up pneumoperitoneum and maintained the pressure at 13–15 mmHg, 10 mm trocars were placed in the umbilicus and subxiphoid process, respectively. 5 mm trocars were placed in the middle of the right clavicle and the right anterior axillary line, respectively. After dissection of Calot's triangle, the cystic artery was clipped and cut with coagulation, then the cystic duct was clamped with a 10 mm titanium clip near the gallbladder to prevent gallstones entering the CBD during the procedure, finally the cystic duct was closed and cut. It is recommended that further intraoperative cholangiography be performed for those patients whose choledocholithiasis has not been identified by conventional imaging. A hole was cut in the upper 1/3 of the cystic duct, after removing the internal gas, the C-tube (Fr 5 ureteral catheter) was inserted through the incision into CBD gradually and the depth was 2.0–2.5 cm. After the bile was withdrawn, it was advisable to fix the cystic duct and C-tube to ensure that there was no obvious resistance and no bile leakag. A C-arm machine was used for intraoperative radiography, 3–5 ml of 17.5% iohexol was injected to observe the morphological feature and patency of CBD, and further determine the presence of CBD stone(s). Subsequently, 5–10 ml of 17.5% iohexol was further injected for observing the morphological characteristics of the intrahepatic and extrahepatic bile ducts. After cholangiography, the cystic duct was closed and cut. When the anterior wall of CBD was fully dissected, a longitudinal incision was made on its surface and a flexible choledochoscope was placed into the CBD through the trocar under xiphoid process, stones were directly removed by a disposable stone basket with choledochoscope. Surgeons confirmed CBD clearance by exploring CBD downward to sphincter of Oddi and upward to the bifurcation of the left hepatic duct and right hepatic duct (9).

**Figure 1:**
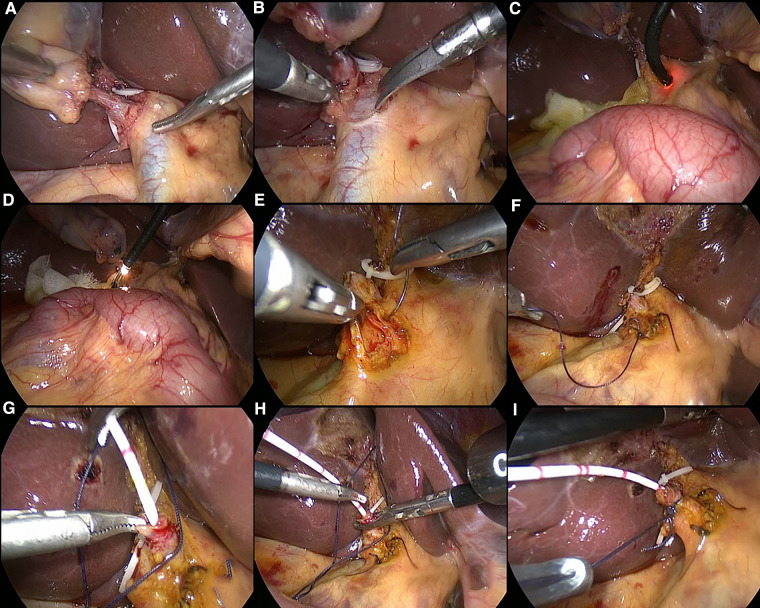
Schematic illustration of laparoscopic choledocholithotomy and C-tube drainage. Figures (**A,B**) show the morphology of the cystic duct and common bile duct; Figures (**C,D**) show the process of choledochoscopy and stone removal; Figures (**E,F**) show the procedure of primary suture of the common bile duct; Figure (**G–I**) show the process of the insertion of C-duct through the cystic duct.

All the patients understood the surgical method and signed the surgical consent form. After removal of the CBD stones, the patients in C-tube group underwent primary suture of CBD with 3-0 absorbable sutures (Johnson, USA), and then C-tube was placed through the cystic duct stump and fixed with a sliding knot. The drainage tube was drawn and fixed under the costal margin of the right abdominal wall. The CBD incision and cystic duct stump were checked for no bile leakage, the procedure was illustrated by Figure 1. For the patients in T-tube group, T-tube was placed in CBD and closed with the same suture material, the drainage tube was also drawn and fixed under the costal margin of the right abdominal wall. Finally, a silicon drainage tube was inserted through the lateral trocar and fixed on the port of right anterior axillary line (9, 11).

### Perioperative management and follow-up

Fasting for 6–8 h preopratively was necessary and oral intake was routinely resumed 12 h postoperatively. If the drainage fluid was <50 ml for 2 days and contained no bile or fresh blood, the silicon tube for drainage would be removed. The C-tube or T-tube was removed within specified time after confirming the absence of remnant stones or stenosis of the CBD by postoperative cholangiogram. In our center, the recommended time for removal of T-tube is 30–40 days and C-tube is 12–15 days. The first outpatient visit was scheduled at 2 weeks after discharge. Imaging studies such as BUS, CT or MRCP would be performed if there were any abnormal findings, the period of follow-up in our study was 6 months (9, 11).

### Statistical analysis

Patients who have undergone primary closure with C-tube drainage were compared with those with T-tube drainage. Results were expressed as mean ± standard deviation and were analyzed with SPSS 26.0 statistical software. Categorical variables were compared between the two groups by using the Chi-square test and Fisher's exact test, while measurement variables were compared by using the student's t test. A *P* value of <0.05 was considered statistically significant.

## Results

To investigate the effectiveness of C-tube drainage with primary closure in the treatment of cholecystolithiasis accompanied with choledocholithiasis. 290 patients underwent LC + LCBDE surgery were enrolled in our study, including 143 cases with C-tube drainage and 147 cases with T-tube drainage. The average age of T-tube group was 64.61 ± 12.92 (years) and C-tube group was 58.89 ± 15.72 (years), there were 75 males, 72 females in T-tube group and 76 males, 67 females in T-tube group. The diameters of CBD were 10.33 ± 2.52(mm) in C-tube group and 10.01 ± 2.48 (mm) in C-tube group. The number and pecentage of obstructive jaundice, acute cholecystitis, acute pancreatitis and acute cholangitis in T-tube group were 70(48%), 80(54%), 12(8.1%), 20(13.6), respectively. The number and pecentage of obstructive jaundice, acute cholecystitis, acute pancreatitis and acute cholangitis in C-tube group were 62(43%), 82(57%), 7(4.9%), 11(7.7). By comparing the personal information and disease information of patients in each group, there were no differences in age (*P* = 0.061), gender (*P* = 0.717), CBD diameters (*P* = 0.279) and some complications between the two groups as follows: obstructive jaundice (*P* = 0.466), acute cholecystitis (*P* = 0.617), acute pancreatitis (*P* = 0.344) and acute cholangitis (*P* = 0.129). All the patients were assessed by American Society of Anesthesiologists scores (ASA), there were 72 patients with ASA1, 70 patients with ASA2 and 5 patients with ASA3 in T-tube group, meanwhile, there were 75 patients with ASA1, 64 patients with ASA2 and 4 patients with ASA3 in C-tube group, meanwhile, the difference was not significant (*P* = 0.825), (All the data was summarized in Table 1).

**Table 1:** Clinical characteristics of patients with LC + LCBDE in each group.

Variable	T-tube group	C-tube group	*P*-Value
	*n* = 147	*n* = 143	
Age (years)	64.61 ± 12.92	58.89 ± 15.72	0.061
ASA scores			0.825^a^
ASA1	72 (49.0)	75 (52.4)	
ASA2	70 (47.6)	64 (44.8)	
ASA3	5 (3.4)	4 (2.8)	
Gender			0.717^a^
Male	75 (51.0)	76 (53.1)	
Female	72 (49.0)	67 (46.9)	
Diameter of CBD (mm)	10.33 ± 2.52	10.01 ± 2.48	0.279
Obstructive jaundice	70 (47.6)	62 (43.4)	0.466^a^
Acute cholecystitis	80 (54.4)	82 (57.3)	0.617^a^
Acute pancreatitis	12 (8.1)	7 (4.9)	0.344^b^
Acute cholangitis	20 (13.6)	11 (7.7)	0.129^b^

Values are presented as mean ± SD or *n* (%).

^a^ Chi-square test.

^b^ Fisher's exact test.

American Society of Anesthesiologists (ASA).

**Figure 2:**
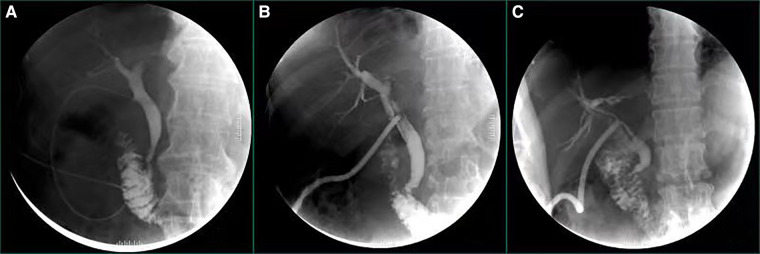
Illustration of postoperative cholangiography, figure (**A**) illustrates postoperative cholangiography *via* C-tube; figure (**B,C**) illustrate postoperative cholangiography *via* T-tubes.

The average time of hospital stay was 11.14 ± 3.63 (days) and postoperative hospital stay was 9.10 ± 3.27 (days) in T-tube group. Compared to T-tube group, the average time of hospital stay and postoperative hospital stay of C-tube group were 9.01 ± 3.49(days) and 7.17 ± 2.10(days), respectively. The patients of C-tube group had shorter in hospital stay, the difference was significant (*p* < 0.001). The time for placing and fixing T-tube intraoperative was 18.59 ± 2.98 (minutes) and the time of removal T-tube postoperative was 31.05 ± 1.90 (days), the time for placing and fixing C-tube intraoperative was 9.83 ± 2.54 (minutes) and the time of removal C-tube postoperative was 11.77 ± 1.40 (days), The time for placing and fixing C-tube intraoperatively and removal C-tube postoperatively was significantly short (*P* < 0.001). The distribution of IOC approach was used almost equally in the 2 groups for confirming common bile duct stones and morphological features of bile ducts, there were 57 patients in T-tube group underwent intraoperative angiography, while 77 patients in C-tube group underwent intraoperative angiography, the number in C-tube group appeared to be higer and the difference was significant (*P* = 0.01). We paid attention to the postoperative biliary drainage situation, therefore, we recorded and statistically analyzed the bilary drainage after the operation. In T-tube group, it was 10.33 ± 3.92 (ml/h) in the first day and 13.27 ± 5.00 (ml/h) in the third day after operation, meanwhile, in C-tube group, it was 9.80 ± 4.50 (ml/h) in the first day and 12.63 ± 5.10 (ml/h) in the third day after operation, There was no significant difference on the rate of bile drainage in the first and third days between 2 groups (day 1 *P* = 0.287; day 3 *P* = 0.279). Nevertheless, within 3 days postoperative, there was an increasing trend in bile drainage in both groups. By comparing the rate of bile drainage on the first and third day after operation in both groups, there were significant differences (*P* < 0.001). (All the data was summarized in Table 2). We can use postoperative cholangiography to further determine the morphological characteristics of the common bile duct, and the presence or absence of residual stones (Figure 2).

**Table 2:** Surgery-related conditions and postoperative outcomes of patients in each group.

Variable	T-tube group	C-tube group	*P*-Value
	*n* = 147	*n* = 143	
Hospital stay (days)	11.14 ± 3.63	9.01 ± 3.49	<0.001
Postoperative hospital stay (days)	9.10 ± 3.27	7.17 ± 2.10	<0.001
Time of placing / fixing tubes (min)	18.59 ± 2.98	9.83 ± 2.54	<0.001
time to removal of drainage tube (days)	31.05 ± 1.90	11.77 ± 1.40	<0.001
Intraoperative Cholangiography	57 (38.8)	77 (53.8)	0.010^a^
Bile drainage (ml/h, day 1)	10.33 ± 3.92	9.80 ± 4.50	0.287
Bile drainage (ml/h, day 3)	13.27 ± 5.00	12.63 ± 5.10	0.279
*P*-value (bile draniage of different day)	<0.001	<0.001	

Values are presented as mean ±SD or *n* (%).

^a^ Chi-square test.

As shown in Table 3, abdominal infection and skin and soft tissue infection appeared to be the most common complications of T-tube drainage, there were 9 paitients with abdominal infection and 23 patients with skin and soft tissue infection. In contrast, patients in C-tube group did not suffer from abdominal infection and skin and soft tissue infection. The differences of abdominal infection and skin and soft tissue infection between the 2 groups were significant (*P* = 0.003 and *P* < 0.001). And let's review the occurrence of other complications. There were 4(2.7%) patients with bile leakage, 2(1.4%) patients with postoperative cholangitis, 2(1.4%) patients with recurrence of bile stones, 1(0.7%) patient with postoperative pancreatitis and no patient with bile duct stenosis in T-tube group, and there were 1(0.7%) patient with postoperative cholangitis, 1(0.7%) patient with bile duct stenosis, 1(0.7%) patient with postoperative pancreatitis and no patient with bile leakage and recurrence of bile stones in C-tube group. There were no significant differences observed between two groups in the rate of bile leakage, postoperative cholangitis, recurrence of bile stones, postoperative pancreatitis and bile duct stenosis (all *P* > 0.05). There was no patient suffered from reoperation in both group. In T-tube group, there were 2(1.4%) paitents were readmitted to hospital for complications in 30days after discharge and 1(0.7%) patient died during the period of hospitalization, however, there was no patient readmitted to hospital for complications in 30 days after discharge or died during the period of hospitalization in C-tube group. The differences in incidence of 30-day readmission and mortality were not significant (*P* = 0.498; *P* = 1.000). The postoperative outcomes after LC + LCBDE of the two groups were summarized in Table 3.

**Table 3:** Postoperative complications of patients in each group.

Variable	T-tube group	C-tube group	*P*-Value
	*n* = 147	*n* = 143	
Bile leakage	4 (2.7)	0	0.123^a^
Postoperative cholangitis	2 (1.4)	1 (0.7)	1.000^a^
Bile duct stenosis	0	1 (0.7)	1.000^a^
Recurrence of bile stones	2 (1.4)	0	0.498^a^
Postoperative pancreatitis	1 (0.7)	1 (0.7)	1.000^a^
Abdominal infection	9 (6.1)	0	0.003^a^
Skin and soft tissue infection	23 (15.6)	0	<0.001^a^
Reoperation	0	0	-
Readmition in 30days	2 (1.4)	0	0.498^a^
Mortality	1 (0.7)	0 (0.7)	1.000^a^

Values are presented as number (%).

^a^ Fisher's exact test.

## Discussion

The current innovation is an attempt to make surgery more precise in our center. In the past 20 years, with the development of laparoscopic and endoscopic technology, biliary system surgery has developed by leaps and bounds. Cholelithiasis is one of the most common clinical diseases in general surgery, and 10%–15% of patients suffered with cholelithiasis and choledocholithiasis concurrently (1, 12). The current treatments of gallbladder stones with common bile duct stones include: OC + OCBDE, ERCP + LC, LC + LCBDE. Comparing to OC + OCBDE, ERCP + LC and LC + LCBDE are more minimally invasive. ERCP + LC requires two procedures, while LC + LCBDE is a single-stage surgery (7, 13). The guideline published in 2017 had proposed in the guidelines for the management of CBDS that LCBDE and ERCP were both very successful in removing CBDS, although there is no evidence that LCBDE differs from ERCP in terms of efficacy, mortality or morbidity, LCBDE and ERCP are considered as two equally effective treatment options. we should admit the importance of ERCP for the management of common bile duct stones, however, in our center, we will firstly choose LC + LCBDE to treat the patients with cholelithiasis and common bile duct stones. We also conduct ERCP research on some elderly patients or patients with acute cholangitis. Our original intention is to solve problems simultaneously and reduce the opportunity of Oddi's sphincterotomy and keep Oddi's sphincter in good condition (14). Long-term treatment effects of LC + LCBDE and ERCP still require multi-center researches.

Complications of LCBDE are mainly related to common bile duct resection (biliary leakage) and T-tube use (biliary leakage, tube displacement). Postoperative biliary leakage will cause a serious of sequelae, such as biliary peritonitis, bacterial infection in abdominal cavity, electrolyte imbalance, and even requires reoperation in severe cases (4, 13). In order to avoid postoperative bilary leakage, T-tube is routinely placed for drainage after LCBDE. However, clinical studies have shown that T tube drainage has disadvantages including tube blockage, tube slip and long retention time, even causes infection of the skin or soft tissues. Most importantly, the integrity of the common bile duct is ruined, which will lead to bilary leakage (7, 11, 15). Therefore, it's crucial to choose an appropriate bile drainage method for the patients.

The normal bile duct is a pipeline system that maintains a low pressure state, which is less than 10 cmH_2_0 (16). The pressure of CBD will increased when stones block the common bile duct, which will cause papilloedema and spasm of the sphincter of Oddi as well. When the pressure reaches to 30cmH2O, it inhibits bile secretion and causes liver dysfunction (17, 18). Although LC + LCBDE has been performed in many medical centers currently, there is still no consensus on the placement of biliary drainage tubes (9, 10, 19, 20). Based on the views above, we chosed two different drainage styles after LC + LCBDE in our study, one was T-tube drainage and another was C-tube drainage. Several recently published studies have reported low bile leakage with only primary suture without drainage tube insertion (9, 15, 21). Indeed, to carry out primay suture without placing biliary drainage the is also the target of our center. Considering that the age, physical condition and the function of Oddi's sphincter are different in patients, surgical options may be different as well. C-tube drainage provides us a novel method to help the patients. We also perform primary sutures without drainage for some young patients or the patiants with good function of the Oddi's sphincter. Although C-tube drainage will bring some discomfort to patients, we hope to provide some strategies for the treatment of patients with cholelithiasis and choledocholithiasis through the research. In this study, 290 patients who underwent LC + LCBDE were divided into two groups according to different drainage styles. From the beginning, our study illustrated an increasing trend of the volume of bile drainage in the first 3 days after surgery, regardless of whether it was T-tube or C-tube drainage. This may be related to postoperative dietary adjustment, Oddis' sphincter spasm and papillary edema. The specific mechanism has not yet been confirmed, but it advised the necessity of postoperative bile drainage.

This study demonstrated that patients in C-tube group had shorter time of hospital stay and postoperative hospital stay than T-tube group (*P* < 0.05). In addition, the average time of placing and removal of the drainage tubes was significantly less than those of the T-tube group (*P* < 0.05). This study also showed significant differences in the incidence of postoperative abdominal infection and soft tissue infection in the two groups (*P* < 0.05). But, there were no significant differences between two groups in readmission within 30 day, postoperative stricture, secondary operation, stone recurrence and postoperative mortality. We find that postoperative infections mainly occurred in the T-tube group. In our opinion, firstly, the number of patients in our study was still insufficient, secondly, the infection of the patients in the T-tube group occurred mainly in the skin and soft tissues, which was stimulated by long-term T-tube placement. Further more, compared to T-tube drainage, C-tube drainage has advantages as follows: 1. C-tube drainage is placed through the stump of the cystic duct, which is a natural lumen. Combined with primary suture of the common bile duct, the morphological and functional integrity of the common bile duct is preserved. 2. Post-operative statistics show that the bile drainage rate of C-tube is basically similar to that of T-tube, which rarely causes postoperative bile retention. 3. There is little interference to the abdominal tissues for the slender structure and soft character of C-tube, so the patients have less discomfort. 4. C-tube drainage has shorter retention time.

**Figure 3:**
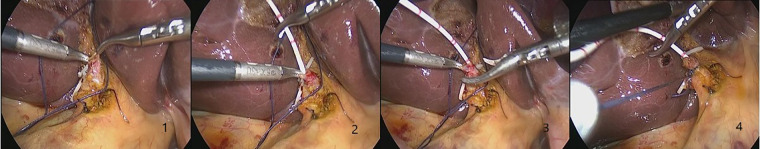
The Figures 1–4 show the process of the insertion of C-duct through the cystic duct. It is necessary to adjust the direction of the cystic duct and maintain proper tension. The first depth of the insertion is about 10.0 cm and is gradually adjusted to 5.0 cm to avoid passing through the Oddi's sphincter. The Duncan knot can ensure the rapid closure of the cystic duct and avoid bile leakage.

This study has also shown that C-tube inserted through the cystic duct had both therapeutic and investigative functions. It not only provided CBD drainage, but also served to inspect the CBD thus avoiding injury and confirming presence of stones. Intraoperative cholangiography through C-tube before choledochotomy could dynamically display the morphological characteristics of bile duct and the contractile function of Oddi's sphincter, which provided more evidence to confirm the diagnosis of choledocholithiasis, and we called it a “supplementary inspection” for MRCP, CT and B-ultrasound. Usually, there are spiral-shaped folds (Heister valves) at the junction between the cystic duct and the common bile duct, which will be the barrier for C-tube insertion (22). Inserting C-tube through cystic duct with Laparoscope is a delicate process that requires proficient surgical skills, therefore, we recommend that this type of surgery should be performed by professional hepatobiliary surgeons, meanwhile, surgeons are encouraged to attend the training of LCBDE to reduce the chances that they may seek for help when dealing with difficult CBD stones (13). Our experiences from this study including (Figure 3): 1. When the surgeon inserts the catheter through cystic duct, it is necessary to adjust the direction of the cystic duct and maintain proper tension to avoid violent manipulation. The depth of the insertion is about 5.0 cm to avoid passing through the odds' sphincter, C-tube drainage will maintain equivalent to the height of the bile column with normal biliary pressure, external drainage will be “automatic” only when the biliary pressure is higher. Under normal circumstances, the bile enters the intestine along the normal channel through Oddi's sphincter, which effectively avoids the loss of a large amount of bile. C-tube can be closed and removed at an early stage. 2. The C-tube is always ligated and fixed with a slip knot (Duncan knot) (23). When tightening the knot, surgeons should not over-tighten it so as to maintain the patency of the drainage tube, however, pipe slippage may be caused by a loose knot. Meanwhile, The Duncan knot can ensure the rapid closure of the cystic duct and avoid bile leakage when the drainage tube is pulled out. 3.After the peak of biliary edema 3 days after operation, patients with C-tube drainage underwent postoperative cholangiography to observe the morphology and function of the biliary tract.

## Conclusion

This study demonstrates a novel method to adjust bile drainage and reduce postoperative complications. Although primary suture of the common bile duct has been studied extensively, postoperative bile drainage is still the key to recovery, especially in acute obstructive suppurative cholangitis (AOSC) and bile duct injury.

## Data Availability

The original contributions presented in the study are included in the article/Supplementary Material, further inquiries can be directed to the corresponding author/s.
